# *Strongyloides stercoralis* disseminated infection in an HIV-infected adult

**DOI:** 10.1371/journal.pntd.0008766

**Published:** 2020-11-05

**Authors:** Ambroise Le Pogam, Julien Lopinto, Adrien Pecriaux, Muriel Fartoukh, Juliette Guitard, Guillaume Voiriot

**Affiliations:** 1 Sorbonne Université, Assistance Publique-Hôpitaux de Paris, Service de Médecine Intensive Réanimation, Hôpital Tenon, Paris, France; 2 Sorbonne Université, Assistance Publique-Hôpitaux de Paris, Service Anatomie et Cytologie Pathologiques, Hôpital Saint-Antoine, Paris, France; 3 Sorbonne Université, Assistance Publique-Hôpitaux de Paris, Service de Mycologie-Parasitologie, Hôpital Saint-Antoine, Paris, France; 4 Sorbonne Université, INSERM UMR S 938, Centre de Recherche Saint Antoine (CRSA), Paris, France; Istituto Superiore di Sanità, ITALY

## Abstract

In this visual case of *Strongyloides stercoralis* disseminated infection with Enterobacteriaceae-related invasive infection, we demonstrated the in-host *S*. *stercoralis* circulation with DNA found in different fluids and specimens, but also in cerebrospinal fluid (CSF), supporting the role of migrant larvae in the Enterobacteriaceae-related invasive and central nervous system infection.

Here, we reported the case of a 66-year-old man who was admitted to our intensive care unit (ICU) for altered mental status. He was living in India but had returned to France for 6 weeks. He had a history of HIV infection and lack of observance of antiretroviral therapies. He was hospitalized because of chronic asthenia, abdominal pain, diarrhea, and fever. Clinic exam revealed periumbilical linear skin lesions of purpuric nature (Fig 1A). Abdominal computed tomography (CT) scan pointed out a thickened jejunal wall, without pneumoperitoneum or peritonitis. He rapidly developed mental confusion with slow and inappropriate verbal responses, leading to ICU admission.

**Fig 1:**
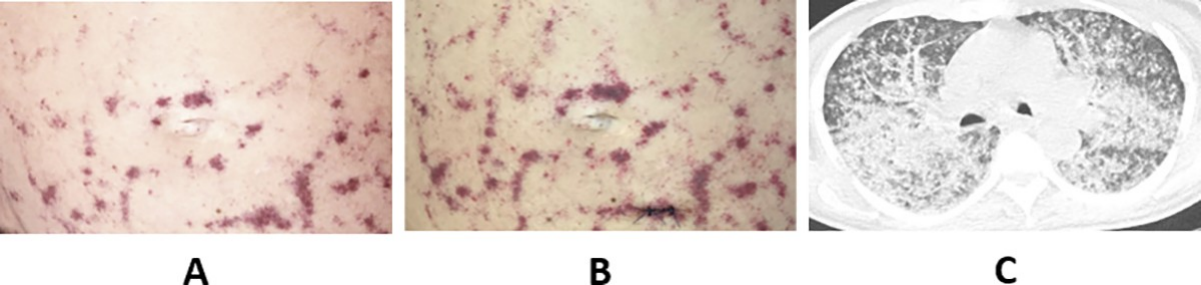
(A) Periumbilical skin lesions at Day 1. (B) Periumbilical skin lesions at Day 2. (C) Multiplanar reconstruction of thoracic CT scan showing bilateral alveolar and interstitial lung infiltrates, with multiple patterns including consolidations, nodules, ground-glass, and tree-in-bud opacities.

At ICU admission, physical examination revealed extensive periumbilical skin lesions (Fig 1B). Brain CT scan did not show any abnormality; however, lumbar puncture revealed a purulent cerebrospinal fluid (CSF). A broad-spectrum antimicrobial therapy was initiated, combined with systemic steroids. Analysis of CSF revealed leukocyte count above 10,000 cells/μL (90% neutrophils), hyperproteinorachia (9.9 g/L), hypoglycorachia (<0.1 mmol/L), and gram-negative bacilli at microscopic examination, leading to dexamethasone interruption. Culture identified cefotaxime-susceptible *Escherichia coli*. On the following day, the patient showed increased hypoxemia and acute respiratory failure, requiring mechanical ventilation. Thoracic CT scan (Fig 1C) pointed out bilateral alveolar and interstitial lung infiltrates, with multiple patterns including consolidations, nodules, ground-glass, and tree-in-bud opacities. Gram stain of bronchoalveolar lavage (BAL) did not reveal any organisms, but microscopic examination revealed larvae of *Strongyloides stercoralis* (Fig 2, S1 Video). BAL cultures grew *E*. *coli*.

**Fig 2:**
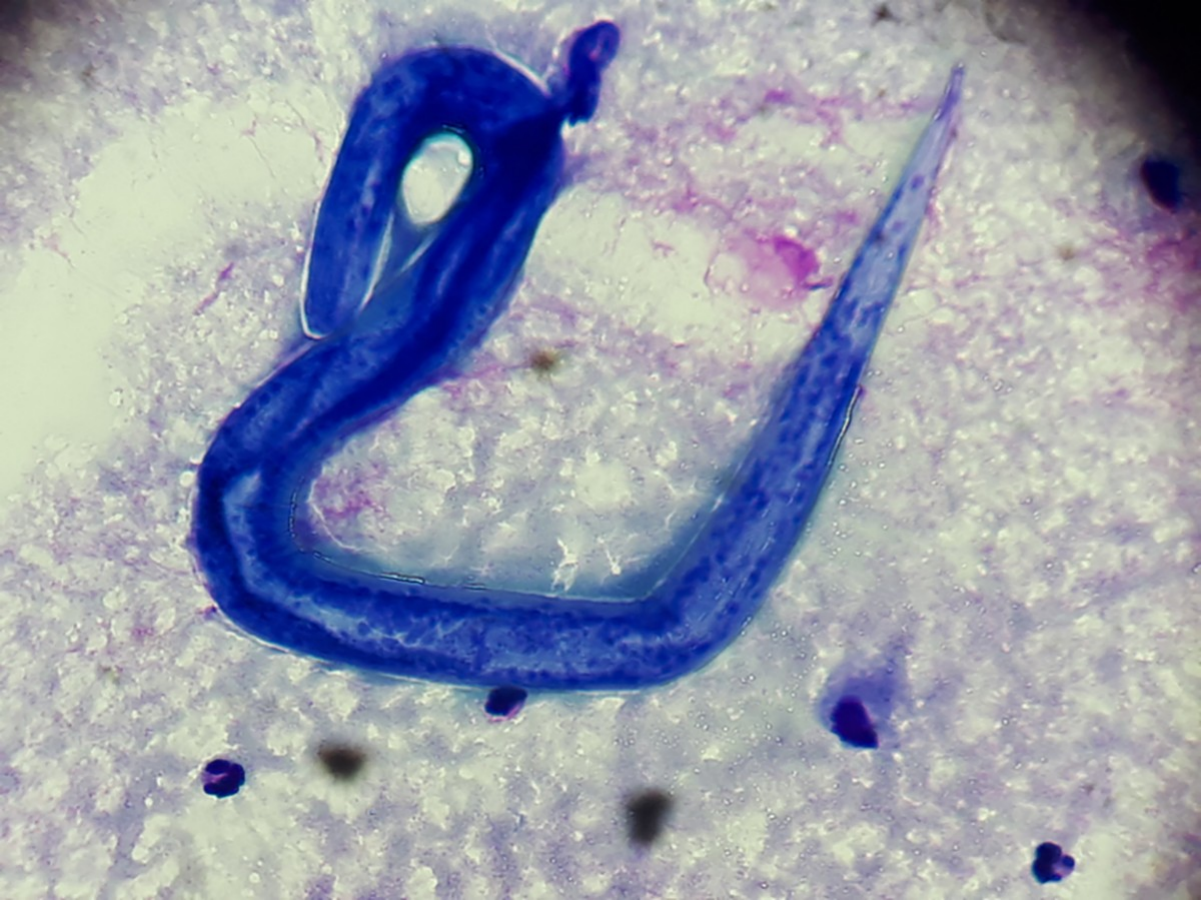
Larva of *Strongyloides stercoralis* in the BAL fluid (Giemsa stain). BAL, bronchoalveolar lavage.

Additional microbiological workup revealed *S*. *stercoralis* larvae in gastric fluid (Fig 3A) and in biopsies from duodenum (Fig 3B) and periumbilical skin (Fig 3C). *S*. *stercoralis* serology (ELISA) was positive. Furthermore, *S*. *stercoralis* DNA was detected with semiquantitative real-time PCR [1] in CSF, BAL, serum, and biopsies from duodenum, stomach, esophagus, and bone marrow (Table 1). Additional microbiological documentations included positive PCR for Cytomegalovirus (blood: 3.6 log copies/mL; BAL: 2.5 log copies/mL; CSF: positive with multiplex PCR), HIV (blood: 5.6 log copies/mL; CSF: 5.6 log copies/mL), and Respiratory Syncytial Virus (BAL: positive with multiplex PCR). Blood culture did not grow *E*. *coli*. The CD4 lymphocyte count was 3 cells/μL. There was no eosinophilia (count: 0.01*10^9^/L).

**Fig 3:**
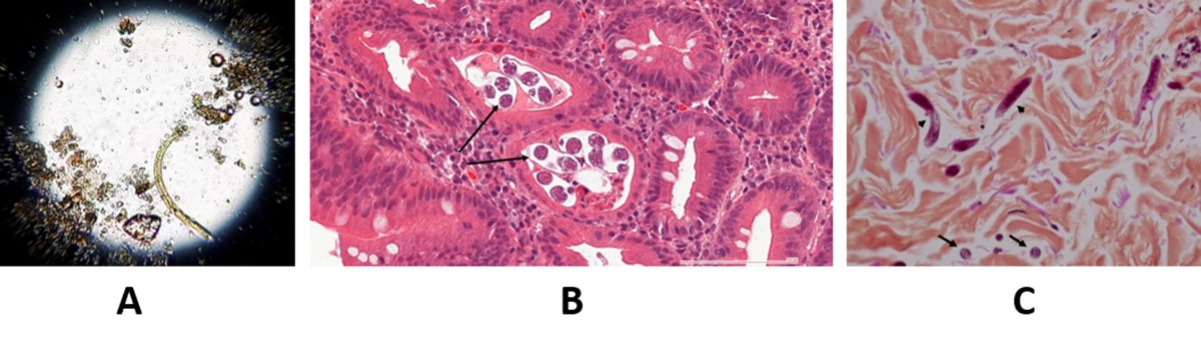
(A) Larva of *Strongyloides stercoralis* in the gastric fluid (direct examination). (B) Histopathologic findings from duodenal endoscopic biopsies, demonstrating in high-magnification (HE stain) reactive epithelium with intraepithelial inflammation (cryptitis) and transversally cut filariform larvae of *S*. *stercoralis* (arrows). (C) Histopathologic findings from skin biopsies, demonstrating in high-magnification (HE stain) transversally cut (arrows) and longitudinally cut (arrows head) filariform larvae of *S*. *stercoralis*. HE, hematoxylin–eosin.

**Table 1:** *Strongyloides stercoralis* DNA detection in different specimens at several timepoints.

Date of sampling	Specimen tested	Cp
D0	CSF	39
D2	BAL	35,6
D3	Stomach biopsy	29
D3	Esophagus biopsy	36
D5	Bone marrow biopsy	40,4
D9	Serum	35
D9	Whole blood	37
D10	BAL	34
D11	CSF	37
D11	Whole blood	38,6

BAL, bronchoalveolar lavage; Cp, PCR crossing point; CSF, cerebrospinal fluid.

In addition to high-dose intravenous cefotaxime and ganciclovir, veterinary parenteral formulation of ivermectin was administered subcutaneously (200 μg/kg q.d. for 2 days). After 5 days, dead larvae were observed in gastric fluid and BAL. The periumbilical skin lesions progressively healed, and the patient recovered from multiple organ failure.

*S*. *stercoralis* disseminated infection has been thoroughly reported in various immunocompromised populations, including patients with hematological malignancies, solid organ transplants recipients, and patients receiving immunosuppressive therapy, especially steroid therapy [2,3]. In HIV-infected patients, disseminated infection has also been described, with or without steroid therapy [2]. In our patient, the only long-lasting factor of immunosuppression was the profound HIV-related T-cell deficiency, as other conditions have been excluded (HTLV1 infection, alcohol consumption, and malnutrition). Pulmonary symptoms are highly frequent, and the detection of larvae in respiratory tract specimens is a hallmark of hyperinfection. Cutaneous periumbilical purpura has been described in patients with disseminated infection, in relation with the migration of larvae through vessel walls to the dermis [4].

Parasitological diagnosis relies on larvae visualization in different specimens. Except in stools where larvae may be highly concentrated, other specimens are often negative with conventional tests. Molecular diagnosis of *S*. *stercoralis* has been developed and validated in stool samples, with very good sensitivity and specificity [1]. Here, we were able to detect DNA even in low parasitic load specimens such as CSF, which was negative with conventional tests.

The association between disseminated *S*. *stercoralis* infection and invasive Enterobacteriaceae infections has been widely reported and is thought to be related to the passive transportation of bacteria on the cuticle of migrating larvae, a phenomenon called phoresis [5]. Here, we demonstrate for the first time the in-host *S*. *stercoralis* circulation. Its DNA was found in different fluids and specimens, but also in a CSF sample, supporting the role of migrant larvae in the Enterobacteriaceae-related invasive and central nervous system infections.

## Ethics

The authors declare that the informed oral consent for publication has been obtained from the patient’s next of kin.

## Supporting information

S1 Video Larva of *Strongyloides stercoralis* moving in the unstained bronchoalveolar lavage fluid. (MOV)
